# Supplementary material for: Diverse roles of the metal binding domains and transport mechanism of copper transporting P-type ATPases
Source: Nat Commun. 2024 Mar 27;15:2690. doi: 10.1038/s41467-024-47001-4 (PMC10973460; doi:10.1038/s41467-024-47001-4)

Diverse roles of the metal binding domains and transport mechanism of copper transporting P-type ATPases

Zongxin Guo[a], Fredrik Orädd[b], Viktoria Bågenholm[a], Christina Grønberg[a], Jian Feng Ma[c], Peter Ott[d], Yong Wang[e], Magnus Andersson[b], Per Amstrup Pedersen[f], Kaituo Wang[a, #,†] & Pontus Gourdon[a,g,†]

[a] Department of Biomedical Sciences, Copenhagen University, Copenhagen, Denmark
[b] Department of Chemistry, Umeå University, Umeå, Sweden
[c] Institute of Plant Science and Resources, Okayama University, Japan
[d] Medical Department of Hepatology and Gastroenterology, Aarhus University Hospital, Skejby, Denmark
[e] College of Life Sciences, Zhejiang University, Zhejiang, China
[f] Department of Biology, University of Copenhagen, Copenhagen, Denmark
[g] Department of Experimental Medical Science, Lund University, Lund, Sweden
[#] Current address: State Key Laboratory of Plant Diversity and Specialty Crops, Institute of Botany, Chinese Academy of Sciences, Beijing, China
[†] Correspondence should be addressed to: K.W. (kaituo@sund.ku.dk) or P.G. (pontus@sund.ku.dk)

Supplementary figure 1

a

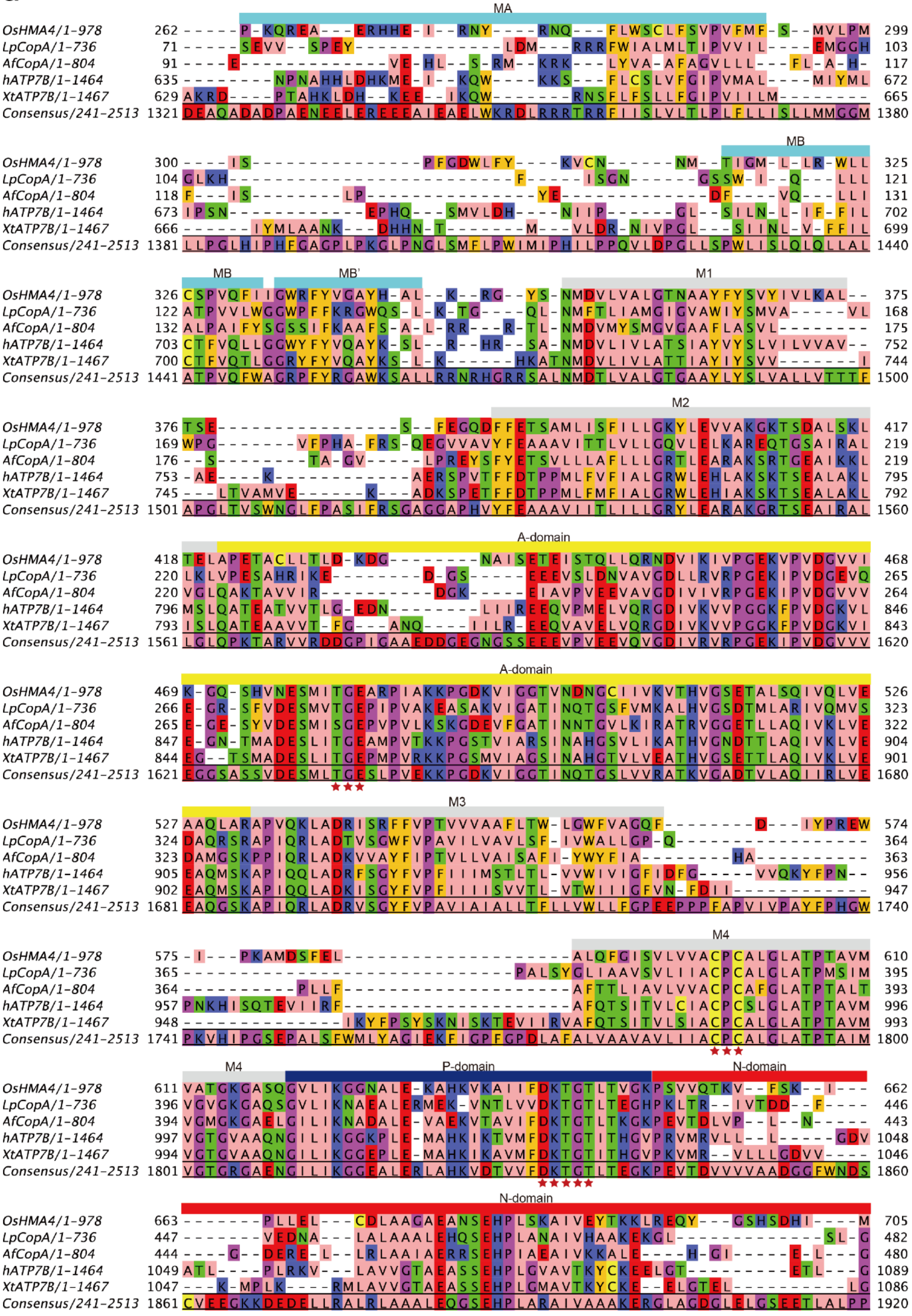

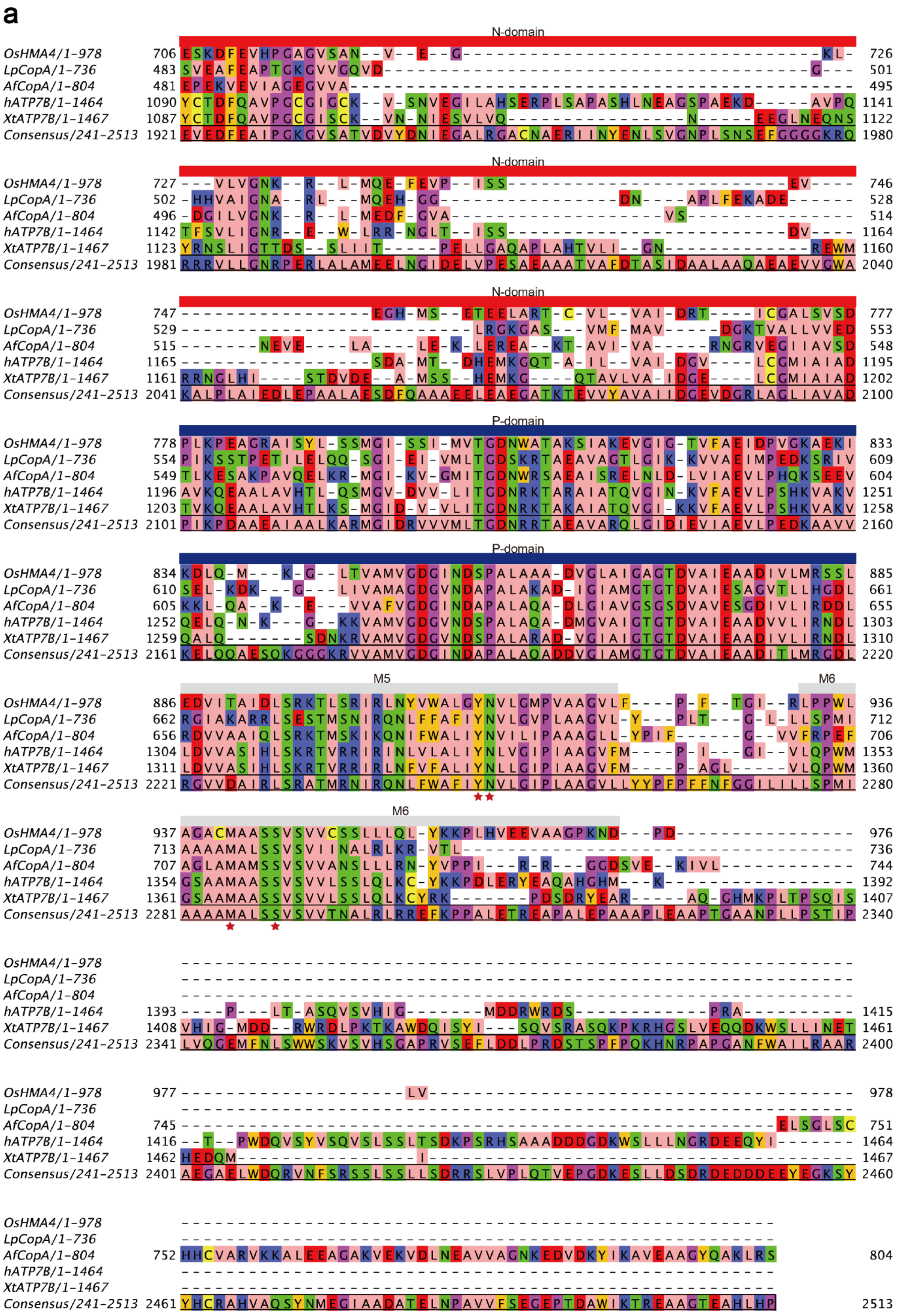
a
N-domain
P-domain
M5
M6
OsHMA4/1-978
LpCopA/1-736
AfCopA/1-804
hATP7B/1-1464
XtATP7B/1-1467
Consensus/241-2513

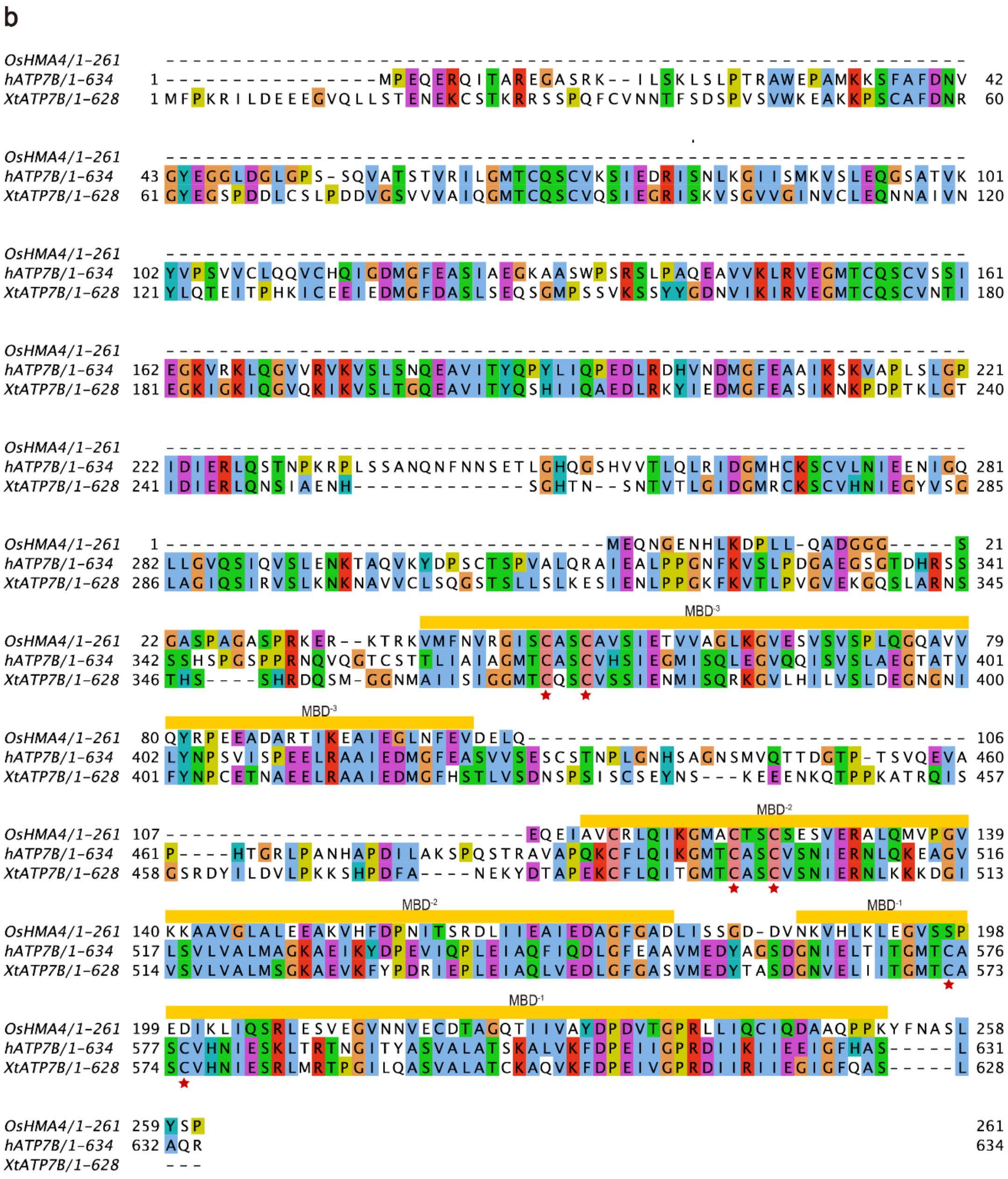

Supplementary figure 1: Conserved sequence features among P_{1B-1}-ATPases. Sequence alignment of copper-transporting P-type ATPases (all containing the CPC motif in M4, DKTGT in the P-domain, YN in M5, and MXXSS in M6 - motifs associated with Cu^+/Ag^+ pumps). Alignments were performed using Clustal Omega and visualized using Jalview. Relevant structural features are highlighted above the sequences. **a,** Alignment of *Oryza sativa* HMA4 (Q6H7M3), *Legionella pneumophila* CopA (Q5ZWR1), Archaeoglobus fulgidus CopA (O29777), human ATP7B (P35670) and *Xenopus tropicalis* ATP7B (A0A6I8R0A5) and the consensus of 1713 CopA proteins, spanning from the MA until the C-terminus (the N-termini have been removed for clarity). **b,** Alignment of the N-termini (until MA) of HMA4, hATP7B and XtATP7B.

Supplementary figure 2

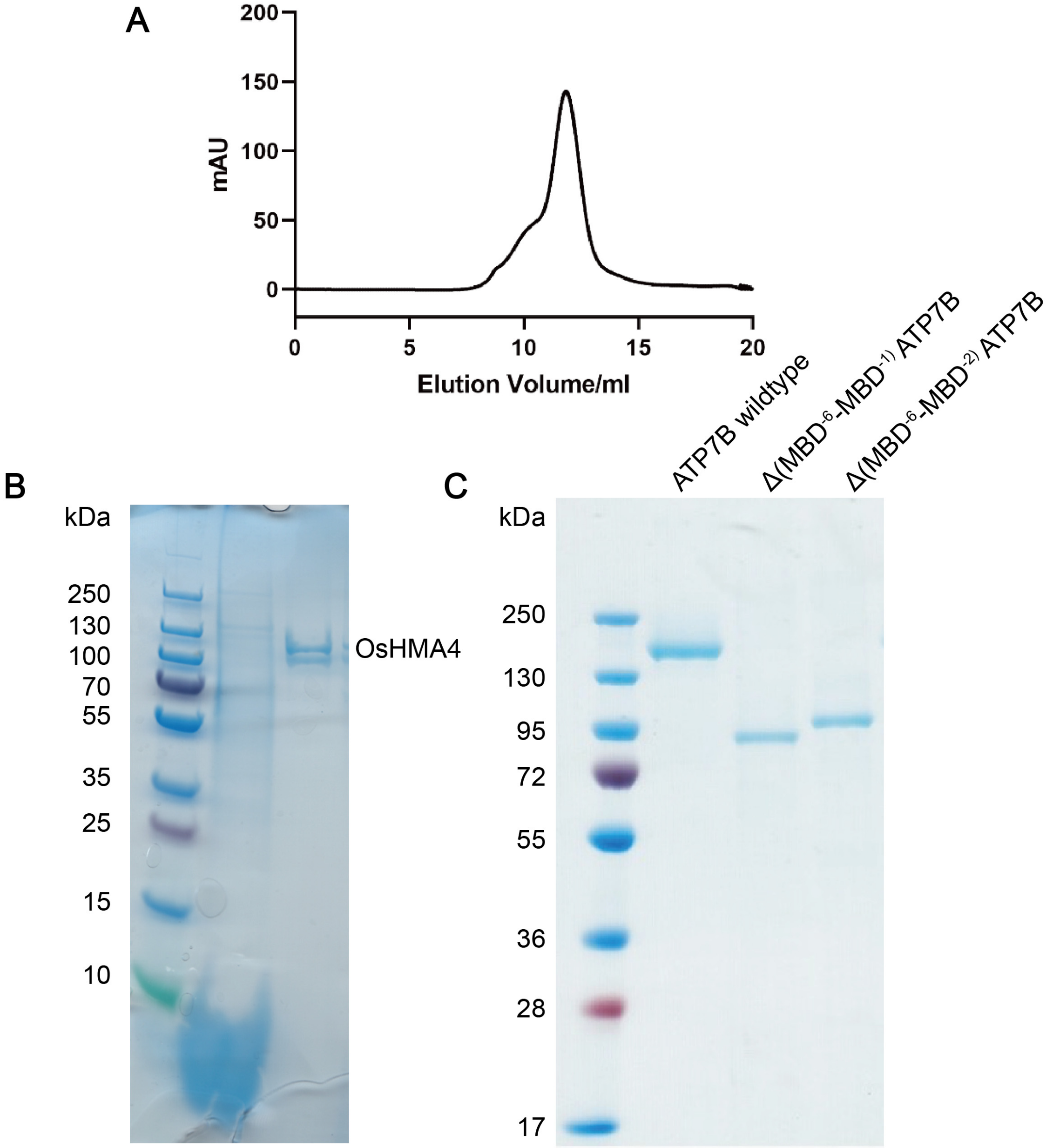

Supplementary figure 2: *Oryza sativa* HMA4 and human ATP7B sample quality as recovered from *Saccharomyces cerevisiae*. a, The size-exclusion chromatography profile following purification of HMA4. **b,** SDS-PAGE analysis of HMA4 following size-exclusion chromatography of HMA4. An unrelated sample is present in the well in-between the markers and OsHMA4. **c,** SDS-PAGE analysis of human ATP7B.

Supplementary figure 3

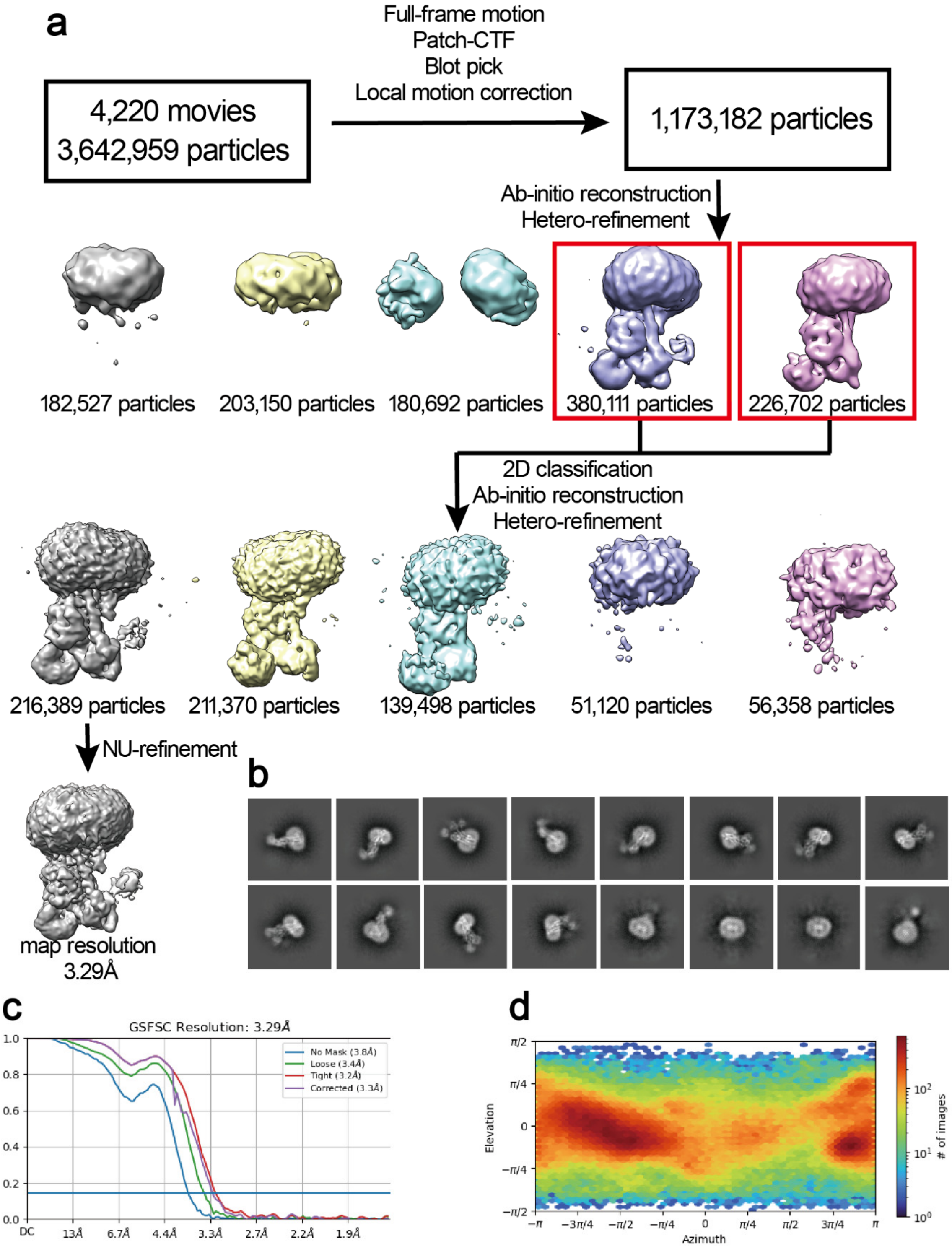

Supplementary figure 3: Data processing of the HMA4^{BeF} structure (E2P state). a, Data processing flowchart. **b,** Representative 2D class averages. The box size was 30 nm. **c,** Gold standard Fourier shell correlation (FSC) curve of the final map. **d,** Particle orientation distributions in the final 3D reconstruction.

Supplementary figure 4

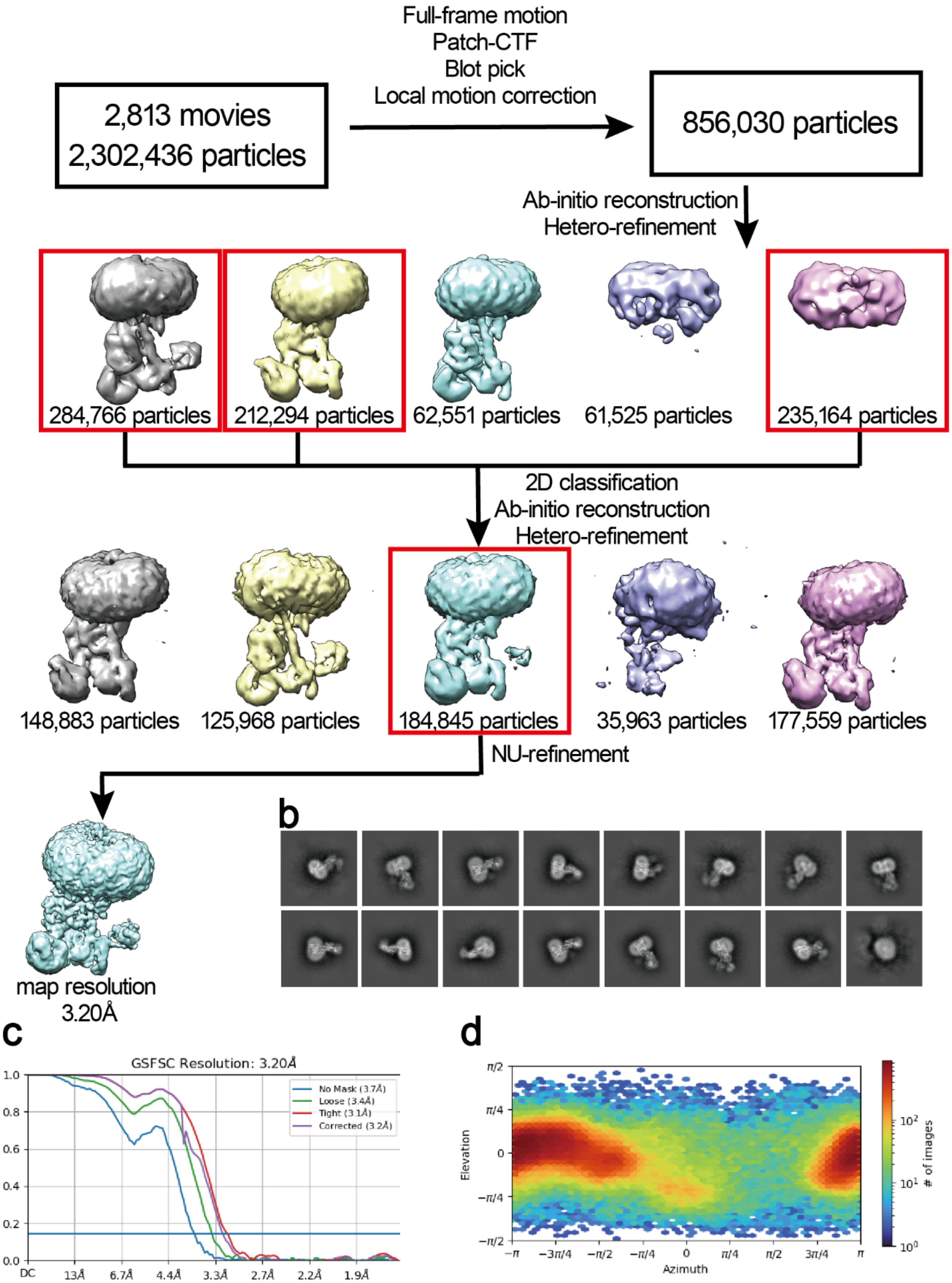

Supplementary figure 4: Data processing of the HMA4^{AlF} structure (E2P state). a, Data processing flowchart. **b,** Representative 2D class averages. The box size was 30 nm. **c,** Gold standard Fourier shell correlation (FSC) curve of the final map. **d,** Particle orientation distributions in the final 3D reconstruction.

Supplementary figure 5

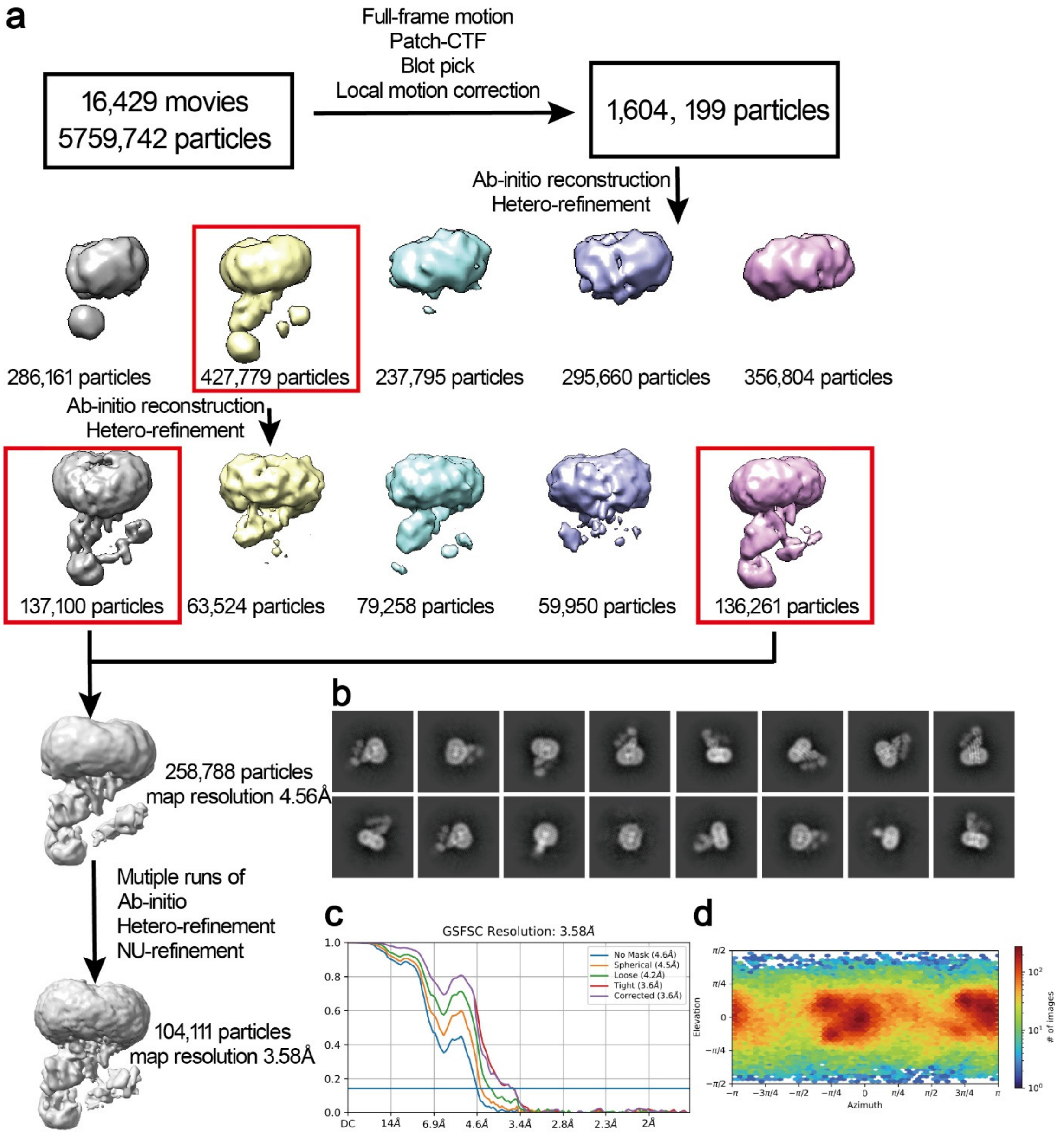

Supplementary figure 5: Data processing of the HMA4^{apo} structure (E1 state). a, Data processing flowchart. **b,** Representative 2D class averages. The box size was 30 nm. **c,** Gold standard Fourier shell correlation (FSC) curve of the final map. **d,** Particle orientation distributions in the final 3D reconstruction.

Supplementary figure 6

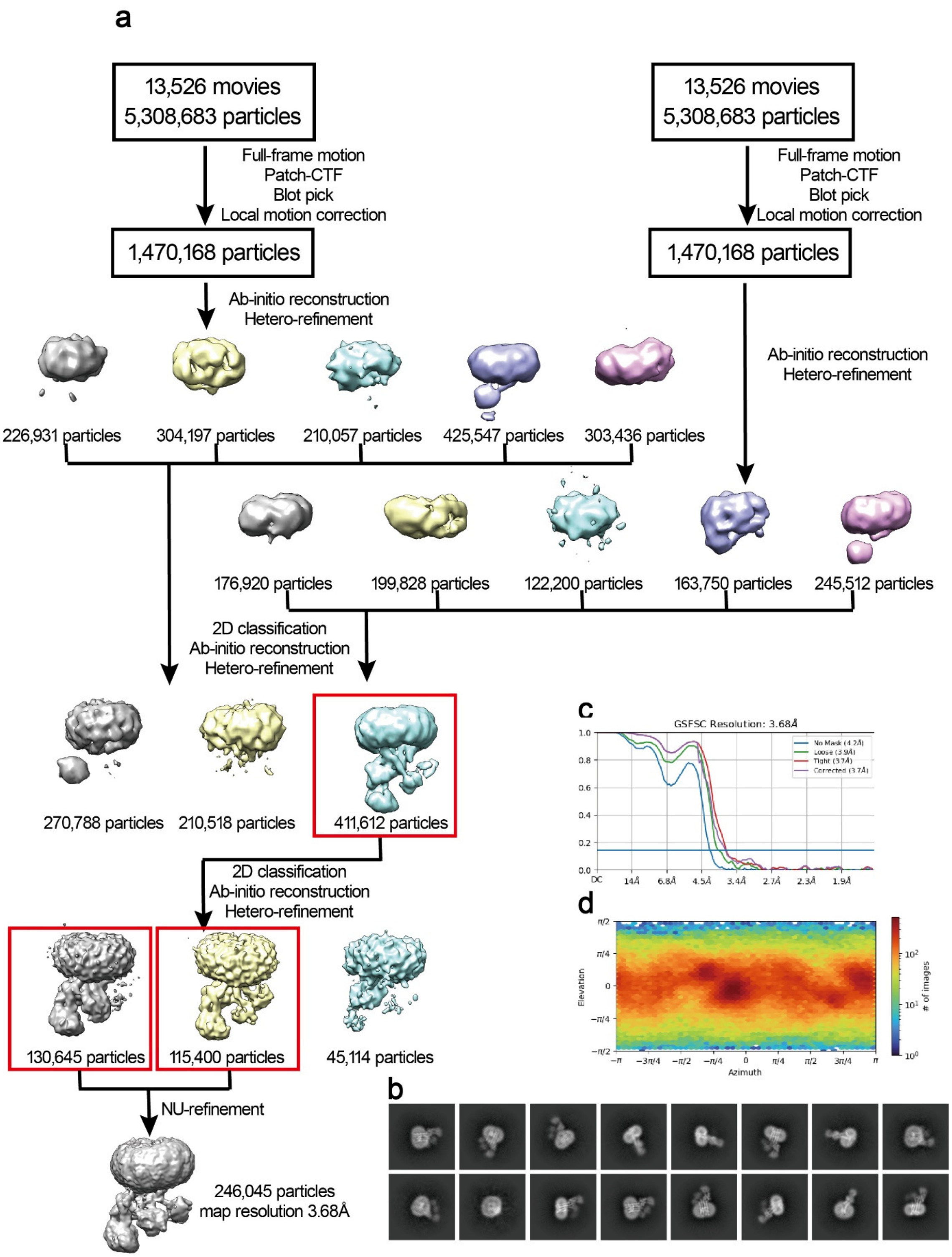

Supplementary figure 6: Data processing of the HMA4^{Cu} structure (E1-Cu state). a, Data processing flowchart. **b,** Representative 2D class averages. The box size was 30 nm. **c,** Gold standard Fourier shell correlation (FSC) curve of the final map. **d,** Particle orientation distributions in the final 3D reconstruction.

Supplementary figure 7

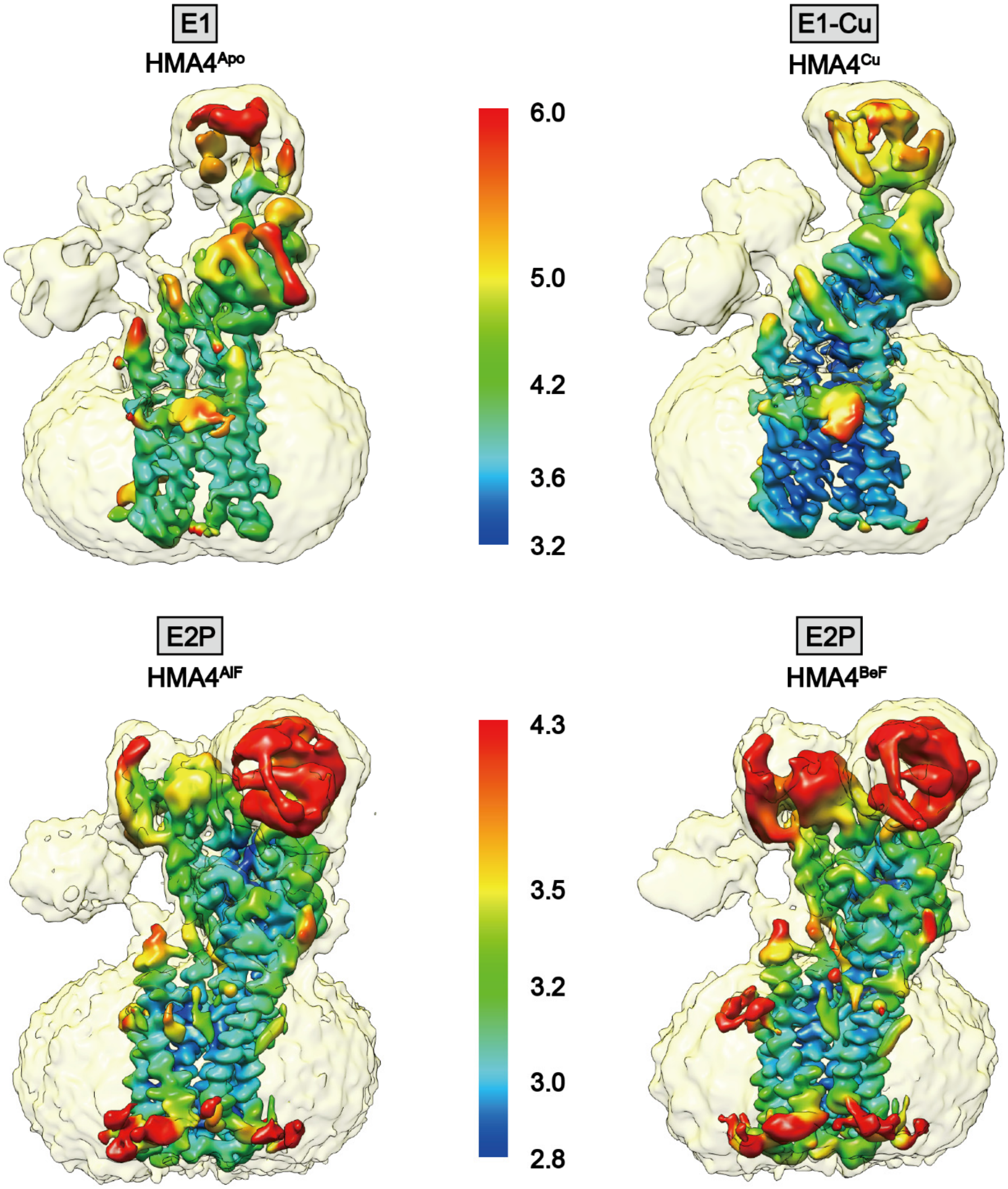

Supplementary figure 7: Local resolution of the cryo-EM maps of the generated HMA4 structures.

Supplementary figure 8

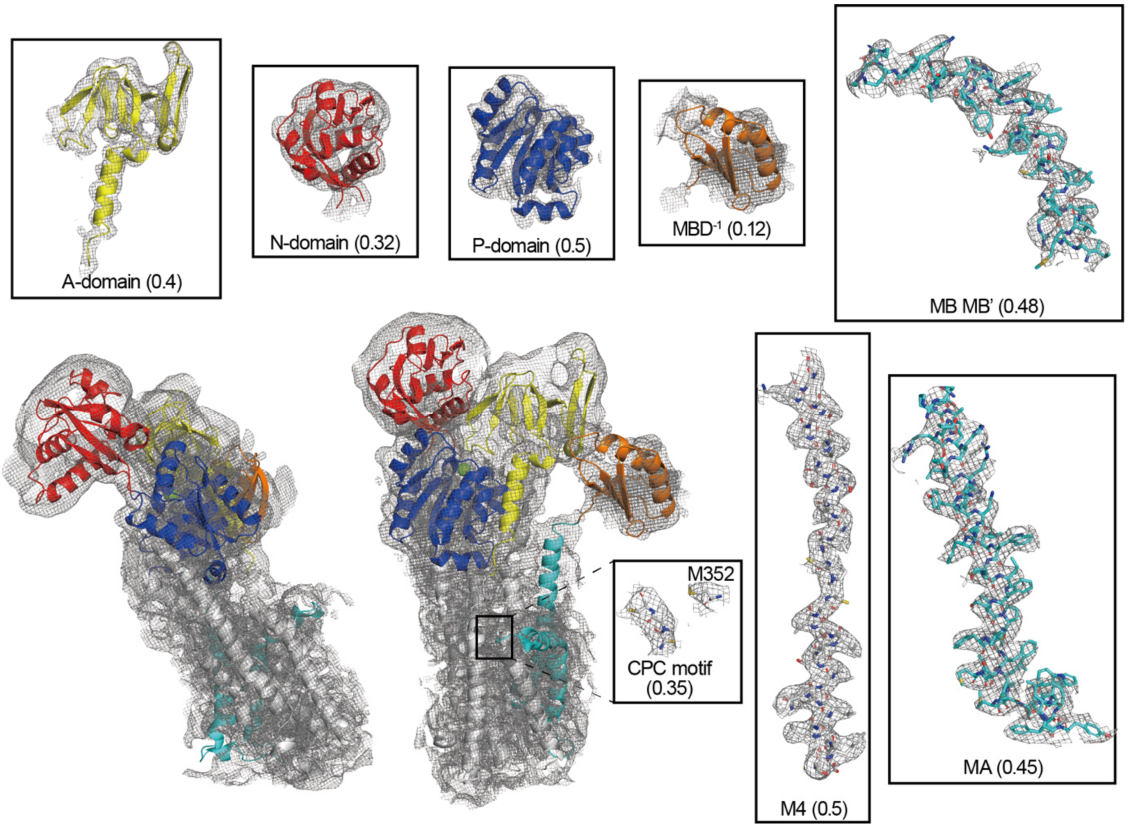

Supplementary figure 8: Cryo-EM map quality of the HMA4^{BeF} structure (E2P state). The protein parts of colored as in Fig. 1. Contour levels are indicated in brackets.

Supplementary figure 9

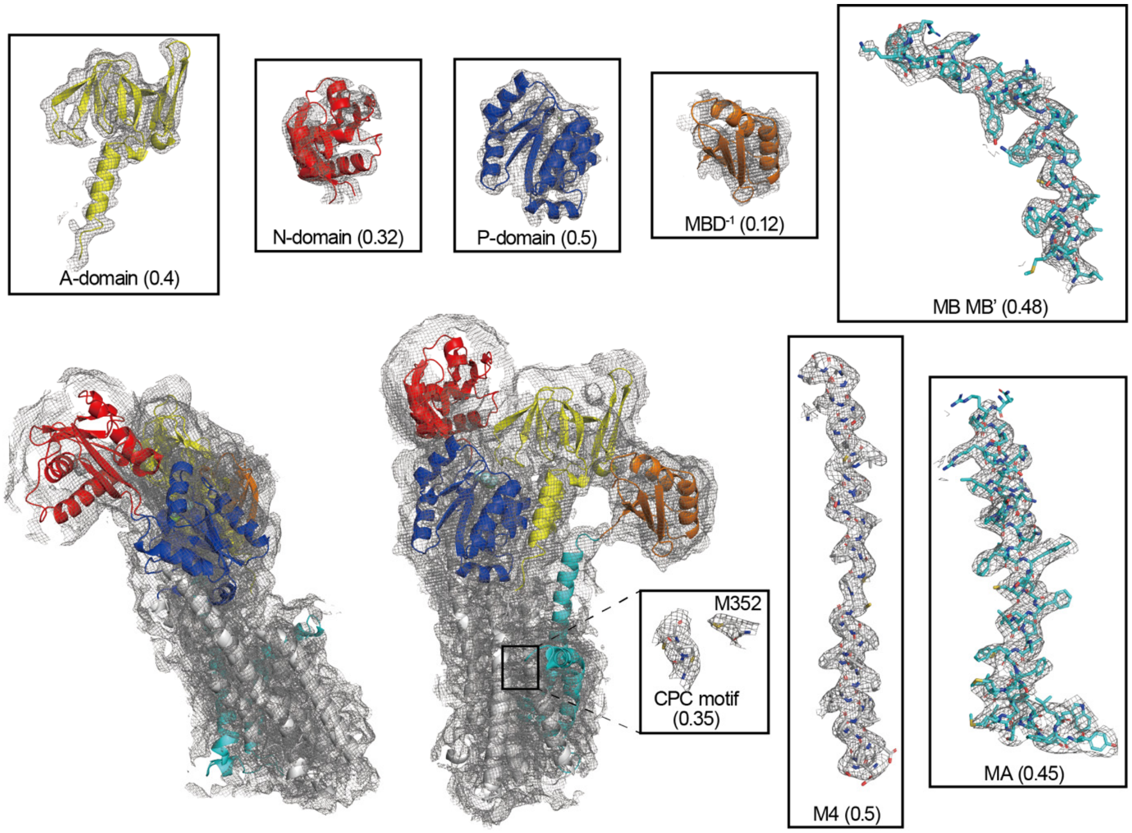

Supplementary figure 9: Cryo-EM map quality of the HMA4^{AlF} structure (E2P state). The protein parts of colored as in Fig. 1. Contour levels are indicated in brackets.

Supplementary figure 10

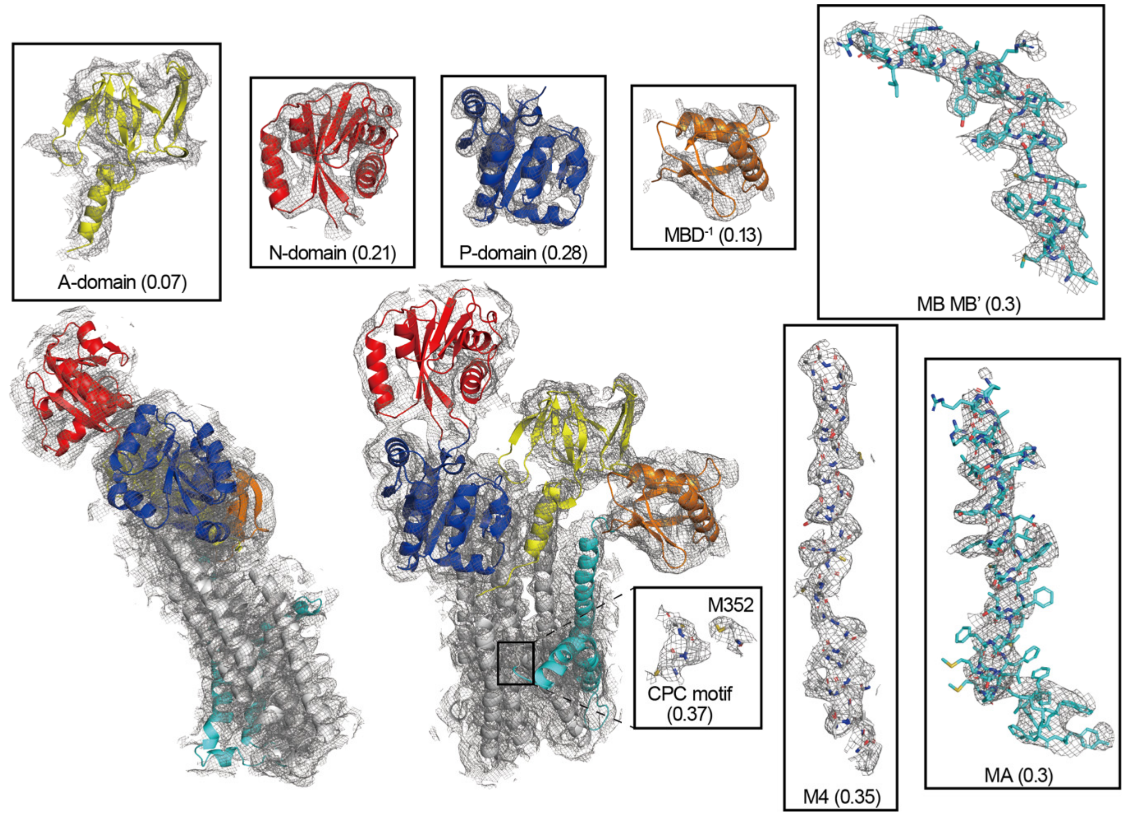

Supplementary figure 10: Cryo-EM map quality of the HMA4^{apo} structure (E1 state). The protein parts of colored as in Fig. 1. Contour levels are indicated in brackets.

Supplementary figure 11

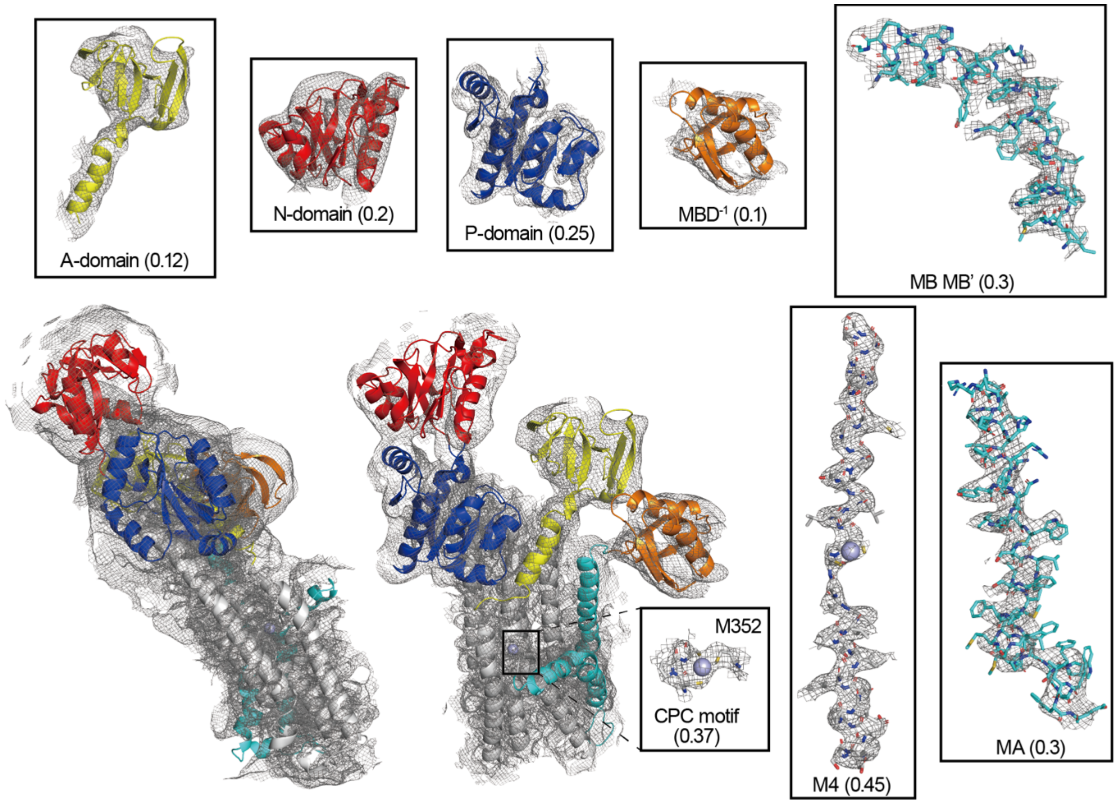

Supplementary figure 11: Cryo-EM map quality of the HMA4^{Cu} structure (E1-Cu state). The protein parts of colored as in Fig. 1. Contour levels are indicated in brackets.

Supplementary figure 12

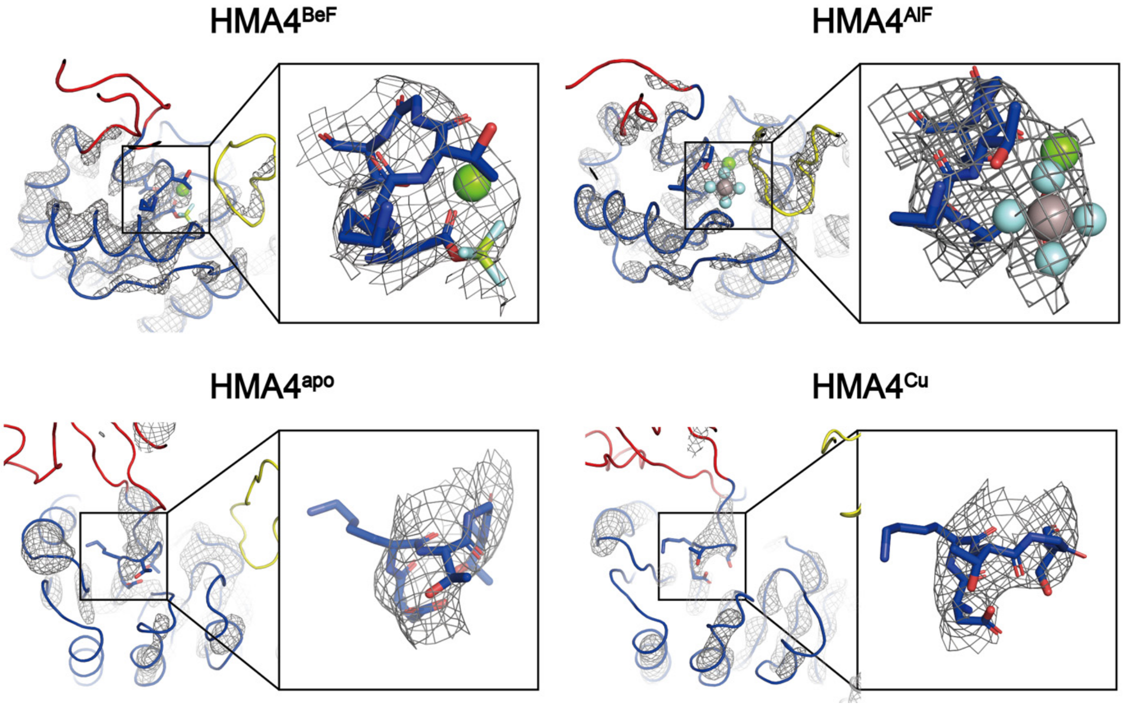

Supplementary figure 12: The DKTGT-regions in the determined HMA4 structures. Note that the density is shown at high signal-to-noise level where even some secondary structure is missing, and yet support for the phosphate mimics is present in HMA4^{BeF} and HMA4^{AlF}, respectively.

Supplementary figure 13

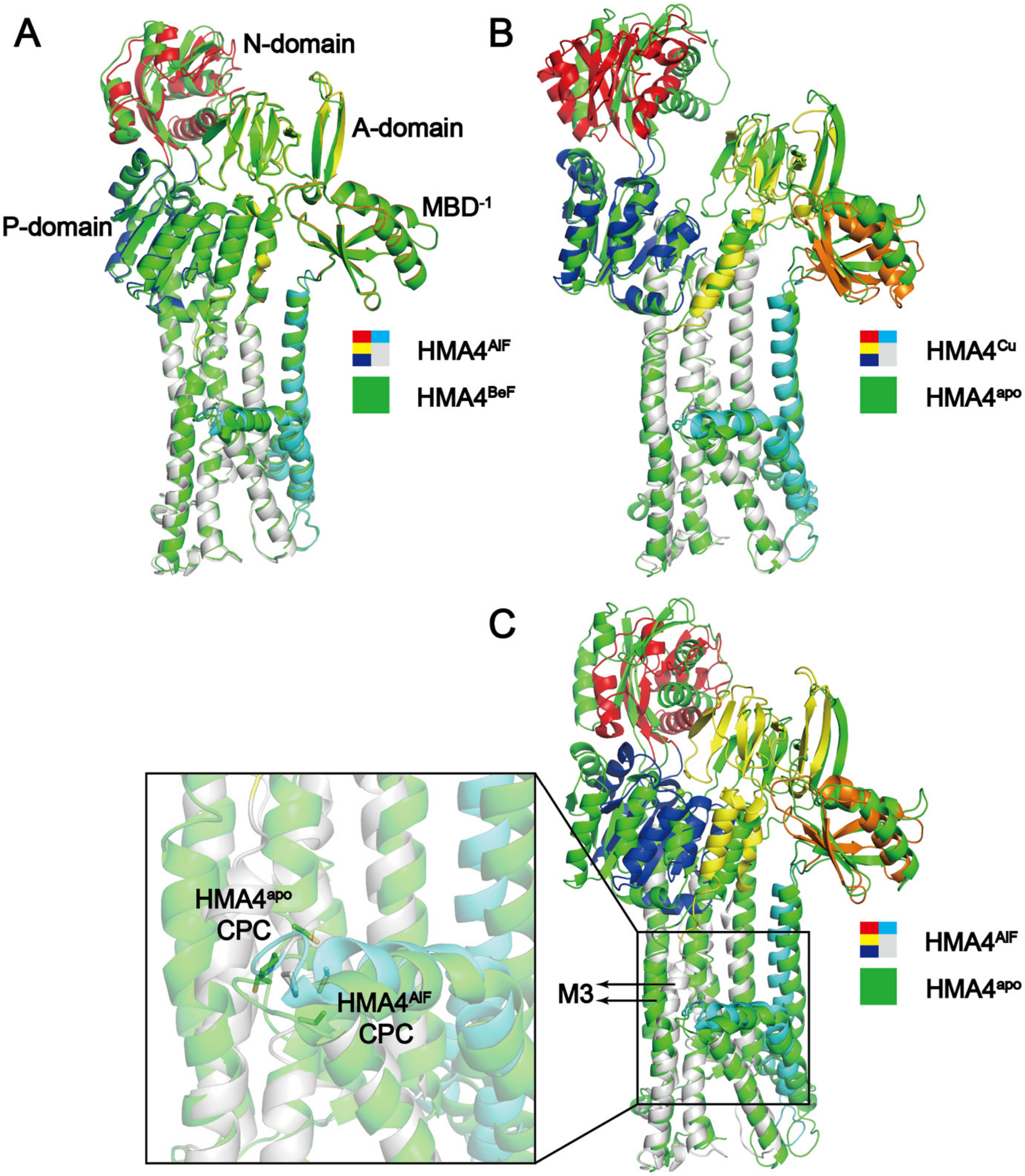

Supplementary figure 13: Structural comparisons of the determined HMA4 structures. Structures were aligned using super in Pymol and using the complete structures. **a,** HMA4^{AlF} versus HMA4^{BeF}. **b,** HMA4^{Cu} versus HMA4^{apo}. **c,** HMA4^{AlF} versus HMA4^{apo}, including a close-view of the CPC-motif showing the CPC-motif is more surface-exposed in HMA4^{apo} than in HMA4^{ALF}.

Supplementary figure 14

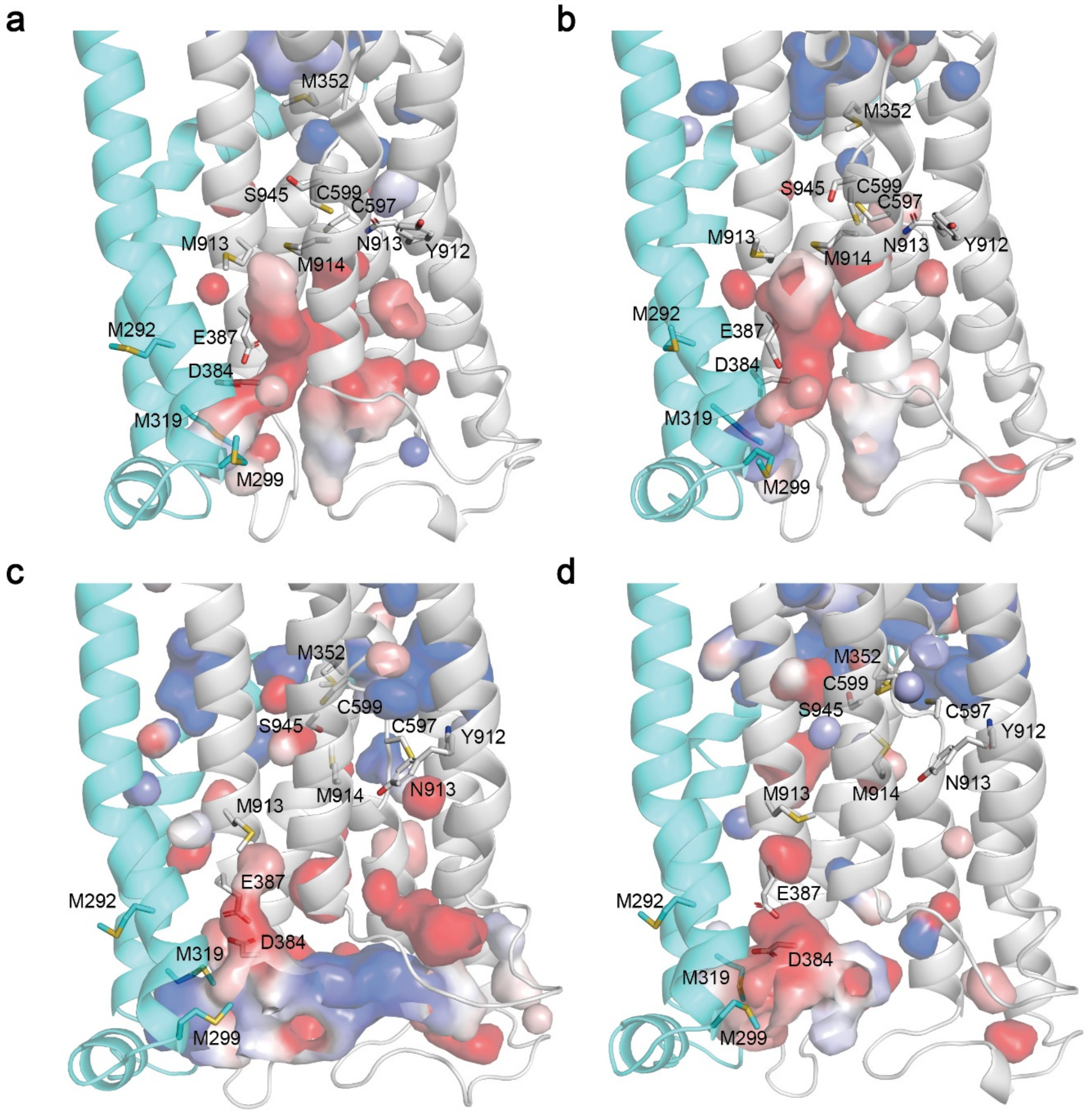

Supplementary figure 14: Surface analyses of HMA4, with access from the extracellular side to the residues contributing to the high-affinity binding site in the E2P, but not the E1 states. a, HMA4^{BeF}; **b**, HMA^{AlF}; **c,** HMA^{apo}; **d,** HMA^{Cu}.

Supplementary figure 15

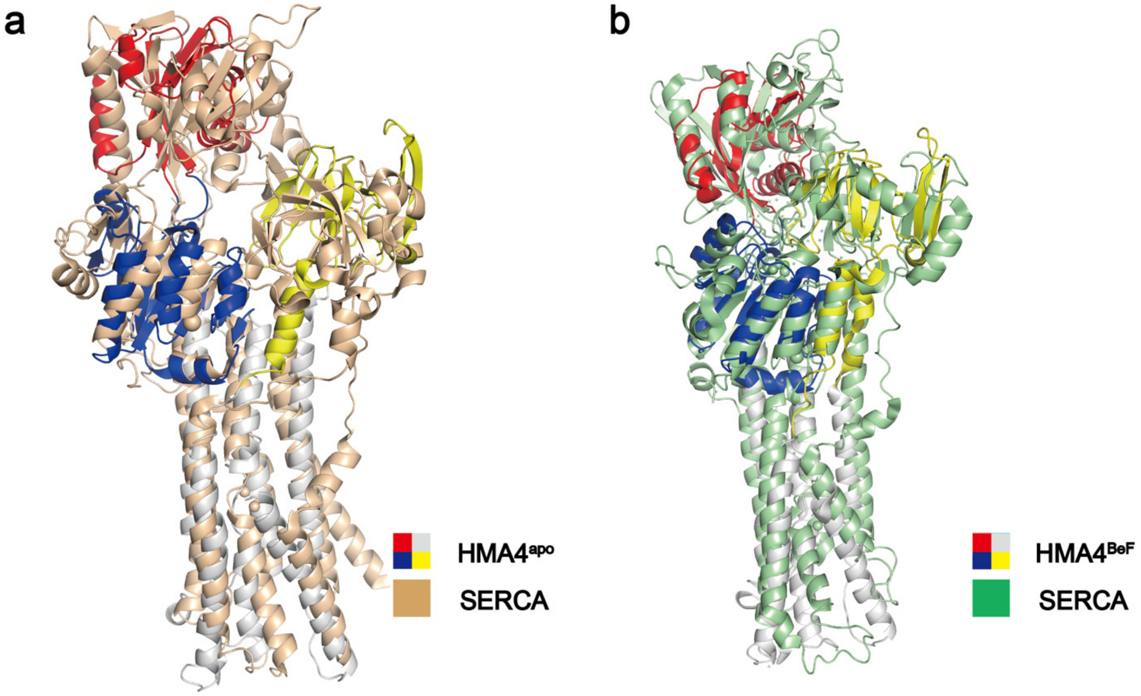

Supplementary figure 15: Structural comparisons of selected HMA4 structures to SERCA. a, Structural comparison of HMA4^{Cu} and SERCA (PDB 4H1W). **b,** structural comparison of HMA4^{AlF} and SERCA (PDB-ID 3B9B). The alignment was conducted using super in Pymol and using the complete structures. For clarity, the MBD, MA and MB as well as M7-M10 have been removed in HMA4 and SERCA, respectively.

Supplementary figure 16

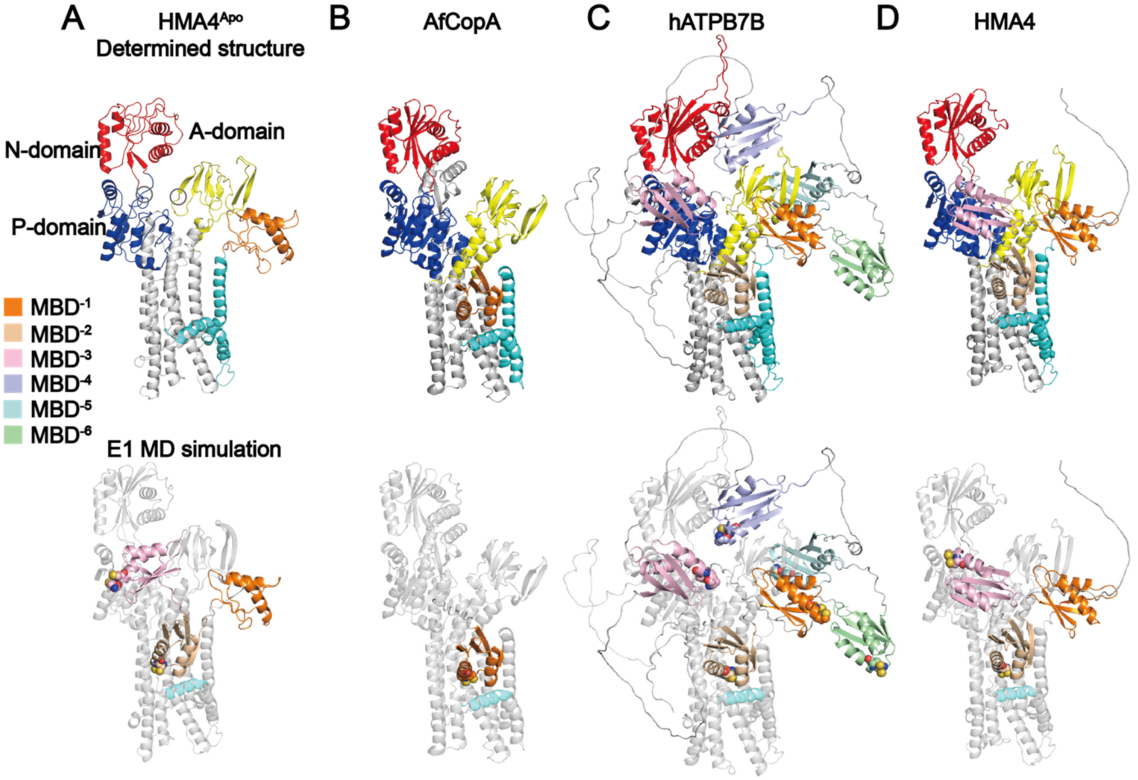

Supplementary figure 16: Comparisons between HMA4apo and AlphaFold structures A, Overview of different P_{IB-I} AlphaFold models, as aligned in PyMol to HMA4apo. Top are overviews of the structures, coloured as in Fig. 1a. Bottom are the same views, but with the core faded out, highlighting the MBDs. The structures shown are HMA4apo (A, including MBDs from the MD simulation in the bottom view), and AlphaFold models of AfCopA (B), hATB7B (C) and HMA4 (D). The MBDs are colored as shown on the left.

Supplementary figure 17

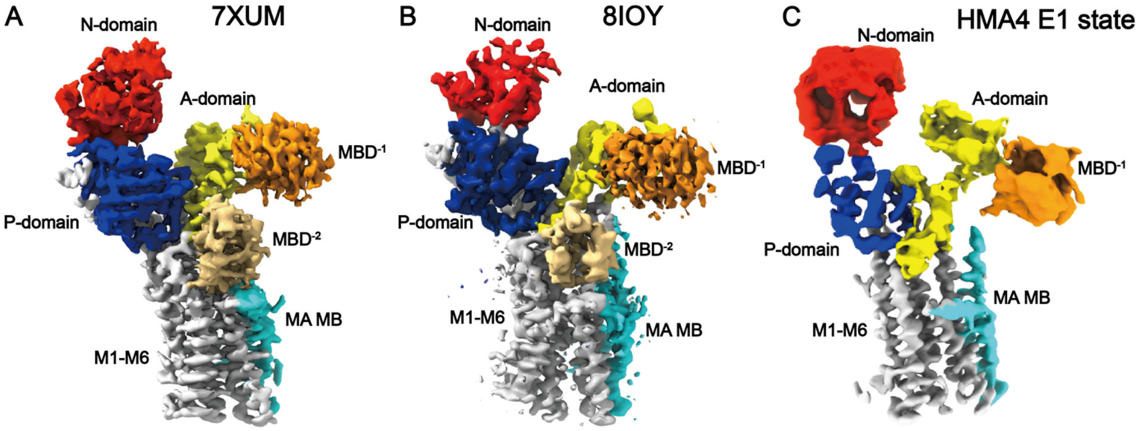

Supplementary figure 17: The previously determined structures of human ATP7B, compared to HMA^{apo}. Density maps of the previously determined structure of human ATP7B (PDB-IDs A: 7XUM B: 8IOY), as well as HMA^{apo} (C). Density of the N-domain in red, P-domain in blue, MA-MB in cyan and M1-M6 in gray. The wheat density was previously assigned as MBD^{-1} while the orange density was left unassigned. We expect the orange density represents MBD^{-1} (as shown in this manuscript), while the wheat density corresponds to MBD^{-2}.

Supplementary figure 18

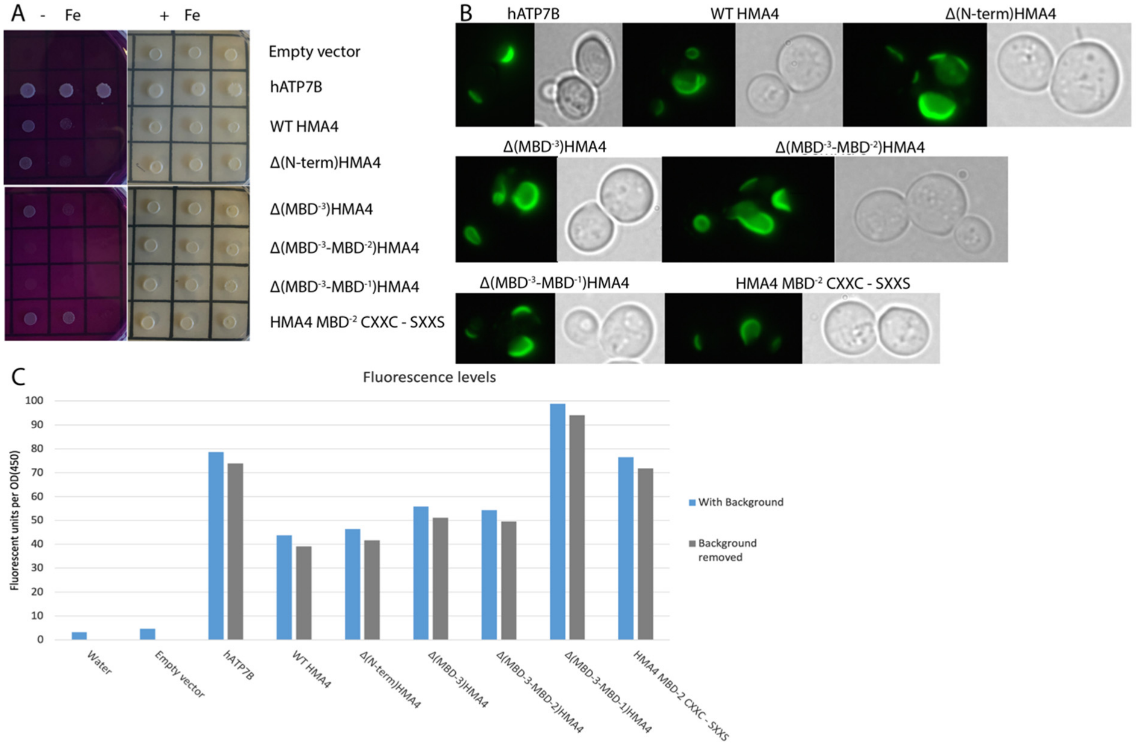

Supplementary figure 18: Complementation of Ccc2p deficient *Saccharomyces cerevisiae*. A: For assessing the function of P_{1B-1}-ATPases, the assay requires low iron levels (-Fe), as Ccc2p is essential for high-affinity iron uptake. Controls grown at normal iron levels (+Fe) are also shown. The indicated yeast strains were spotted at three different densities (from left to right; 5 µL 0.50 OD_{450}/mL; 5 µL 0.05 OD_{450}/mL; 5 µL 0.005 OD_{450}/mL). Plates were imaged after 72 hours. The assessed forms are empty vector (essentially the Ccc2p deficient *Saccharomyces cerevisiae* strain), hATP7B, WT HMA4 (wild-type HMA4), Δ(N-term)HMA4 (HMA4 lacking the N-terminal tail preceding MBD^{-3}), $\Delta(MBD^{-3})$HMA4 (HMA4 lacking MBD^{-3} and preceding amino acids), $\Delta(MBD^{-3}$-$MBD^{-2})$HMA4 (HMA4 lacking MBD^{-2} and all preceding parts), $\Delta(MBD^{-3}$-$MBD^{-1})$HMA4 (HMA4 lacking MBD^{-1} and all preceding parts) and HMA4 MBD^{-2} CXXC - SXXS (HMA4 with the CXXC-motif of MBD^{-2} mutated to SXXS). **B:** Live cell bioimaging of yeast strain PAP6064 expressing C-terminally GFP tagged hATP7B or HMA4 variants. GFP tagged versions of hATP7B, WT HMA4 and HMA4 mutants show similar distinct localization in the *ccc2Δ* yeast strain, probably representing the late-Golgi compartment. Localization in the late-Golgi compartment is required for complementation of the high iron requirement of the *ccc2Δ* strain. Yeast cultures were inoculated at 30 ºC in standard minimal medium with galactose as sole carbon for 24 hours prior to live cell bioimaging. Left side of the figure shows GFP fluorescence while the right side shows the same cells imaged with differential interference contrast. **C:** GFP fluorescence levels detected in yeast strains expressing hATP7b or HMA4 (WT and different constructs).

Supplementary figure 19

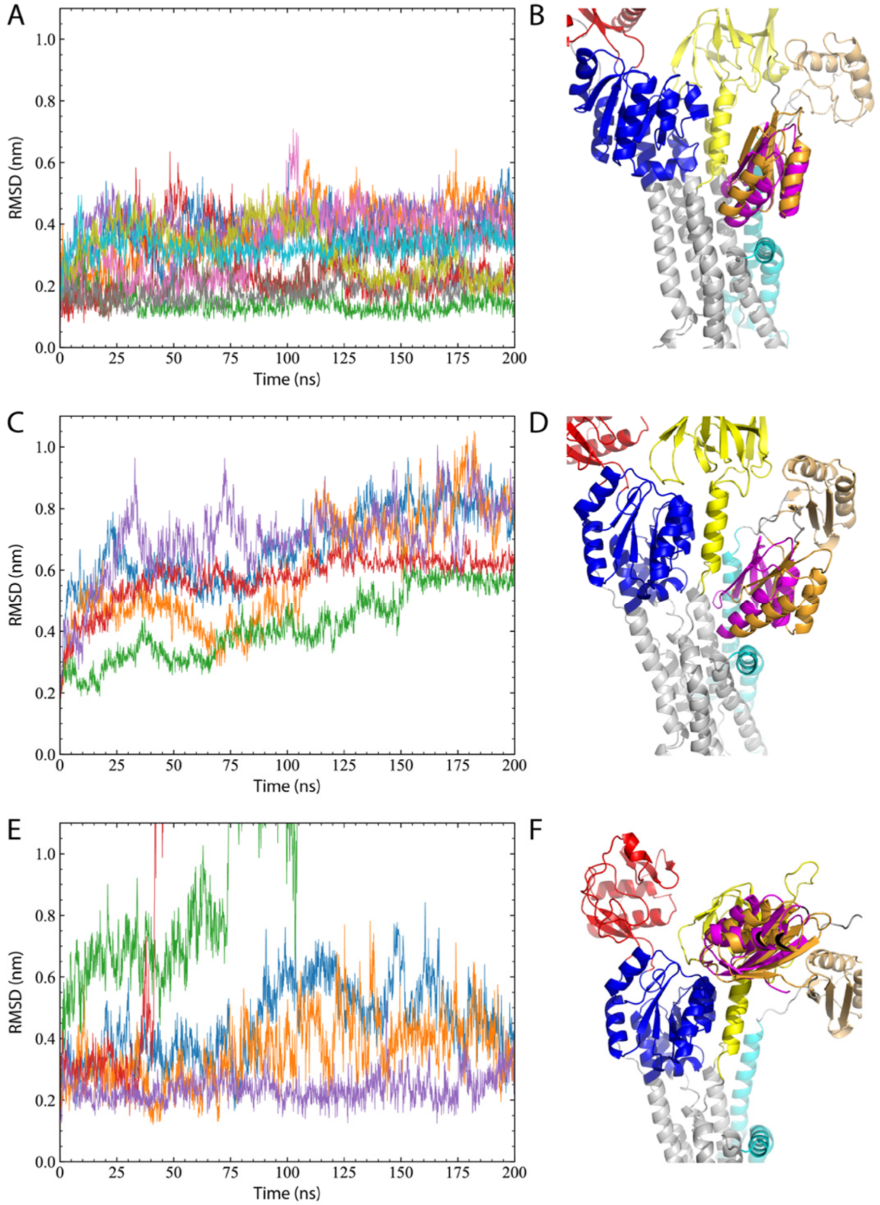

Supplementary figure 19: Molecular dynamics simulations. Stability of MBD^{-2} in the starting position. The RMSD traces of MBD^{-2} against starting structures with MBD^{-2} positioned as in AlphaFold model of HMA4 on MB' in simulations of **a,** the E1 and **c,** E2P state, respectively or **e,** with MBD^{-2} positioned as in the XtATP7B structures (in E2P simulations). The average RMSD with standard deviation for the three systems were 0.30±0.082 nm, 0.60±0.10 nm, 0.98±0.81 nm, respectively. **b,d,f,** shows the final structure of one of the higher RMSD trajectories three separate runs respectively. The M-domain is shown in grey, MA and MB in cyan, P-domain in blue, A-domain in yellow, N-domain in red and MBD^{-2} in orange. The starting position of MBD^{-2} is shown in magenta.

Supplementary figure 20

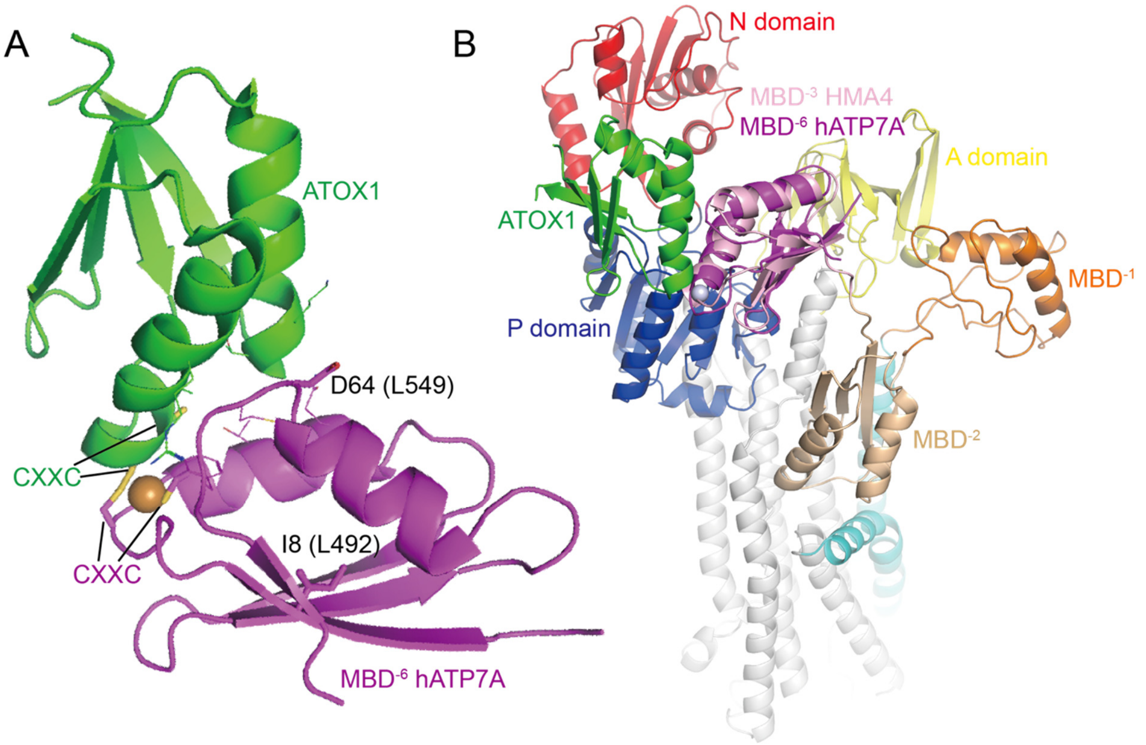

Supplementary figure 20: The complex between ATOX1 and a metal binding domain. A. Structure of ATOX1 in complex with MBD^{-6} of hATP7A (PDB-ID 2K1R). Side chains of residues located at the interaction interface are shown as lines, the CXXC motifs of each domain, as well as I8 and D64 of the MBD (correspond to L492 and L549 of hATP7B MBD^{-2}, respectively, are shown as sticks and labelled. **B.** Structural alignment of the ATOX1-MBD complex (PDB-ID 2K1R) and HMA4 (MD simulation of the E1 state). ATOX1-MBD is colored in green-purple, while HMA4 is colored as in Fig. 3c.

Supplementary figure 21

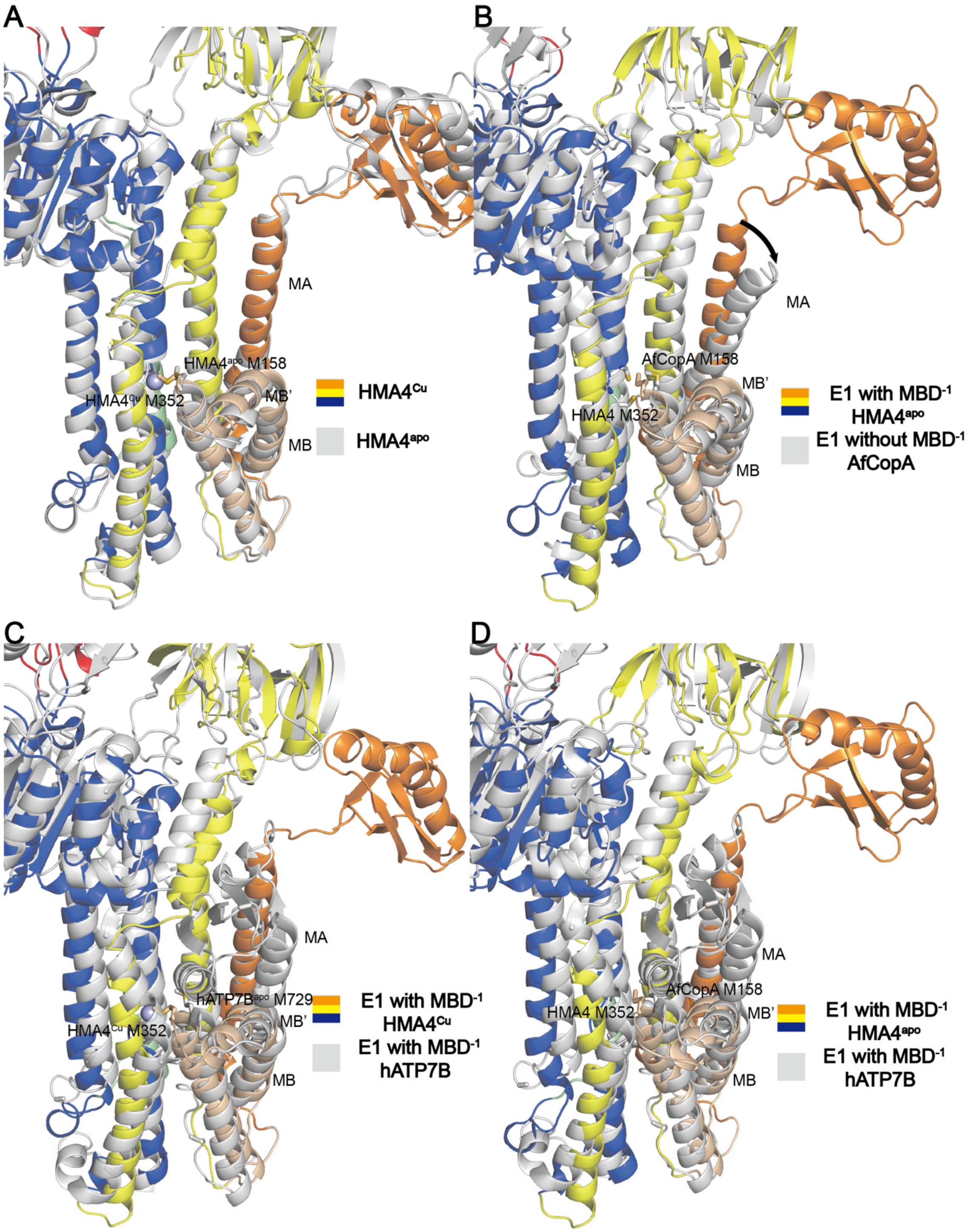

Supplementary figure 21: Structural comparisons of the determined HMA4 structures to other structures of P_{1B-1}-type ATPases.

Supplementary figure 22

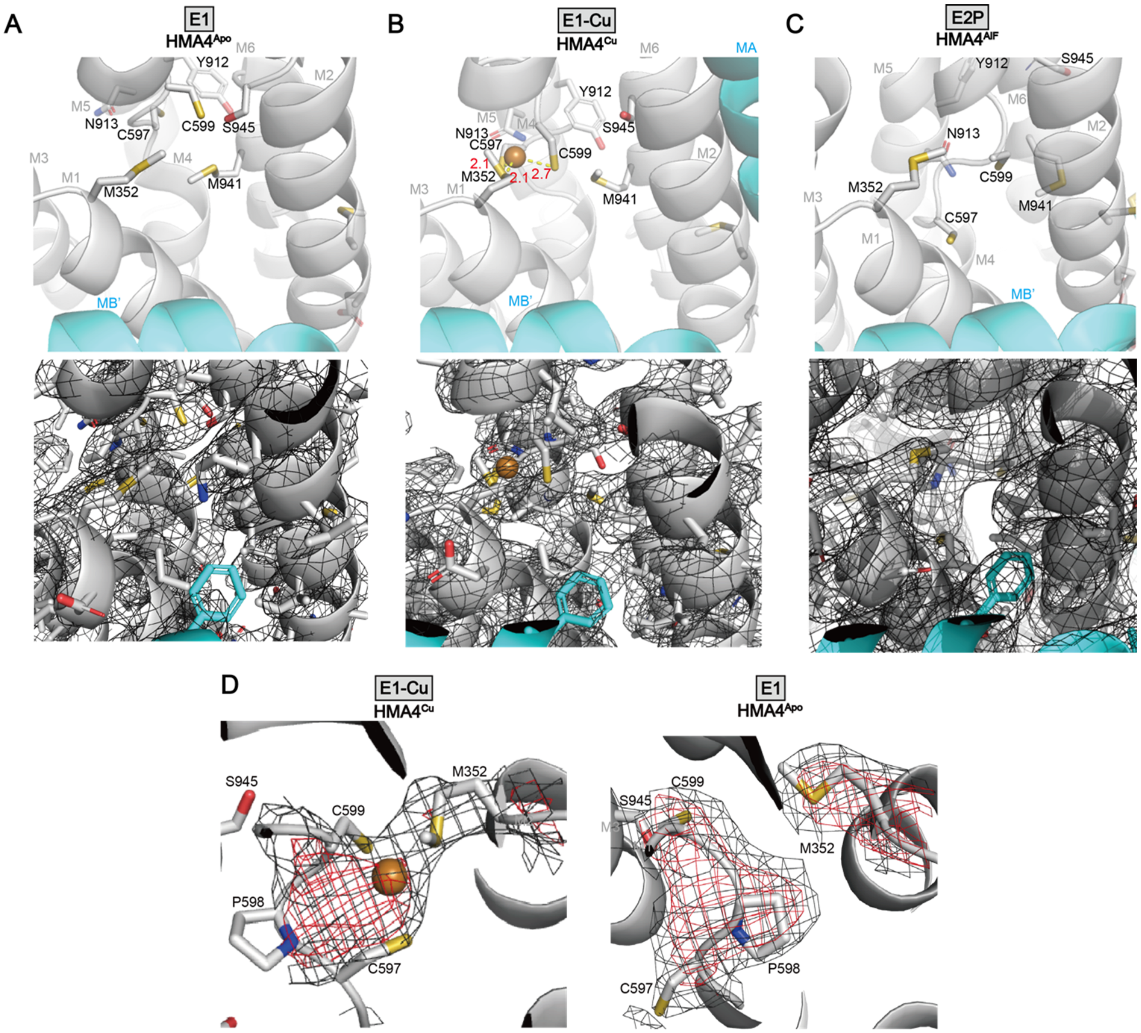

Supplementary figure 22: Densities of the copper binding site A-C, Top are replicates of Fig. 4A-C, with the bottom being the same views including electron density, showing HMA4^{apo} (A), HMA4^{Cu} (B) and HMA4^{AlF} (C). **D.** Closeup of the copper binding site in HMA4^{Cu} (left) and HMA4^{apo} (right), with density shown at two different contour levels in red and black, respectively.

Supplementary table 1

	$HMA4^{apo}$	$HMA4^{Cu}$	$HMA4^{AlF}$	$HMA4^{BeF}$
	**PDB: 8Q73**	**PDB: 8Q74**	**PDB: 8Q75**	**PDB: 8Q76**
**Data collection**				
EM equipment	FEI Titan Krios	FEI Titan Krios	FEI Titan Krios	FEI Titan Krios
Voltage (kV)	300	300	300	300
Detecter	Gatan3	Gatan K3	Falcon 3	Falcon 3
Data collection mode	counting	counting	counting	counting
Pixle size (Å)	0.8617	0.8617	0.832	0.832
Energy filter	/	/	/	/
Electron dose (e^-/Å^2)	50	50	40	40
Defocus range (mm)	-1.2 ~ -2.6	-1.2 ~ -2.6	-1.2 ~ -2.6	-1.2 ~ -2.6
**Data processing**				
Software	cryosparc	cryosparc	cryosparc	cryosparc
Number of final used particles	104111	246045	184645	216398
Symmetry	C1	C1	C1	C1
Map resolution (Å)	3.58	3.68	3.20	3.29
**Model refinement statistics**				
Total built residues	779	784	774	774
Model-map-fit CC	0.73	0.80	0.84	0.82
**R.m.s.d.**				
bonds (Å)	0.004	0.004	0.004	0.003
angles (°)	0.916	1.004	0.982	0.739
**Molprobity statistics**				
Molprobity score	2.23	2.48	2.45	2.32
**Ramachandran plot**				
Favored (%)	86	83	86	87
Allowed (%)	13	16	12	12
Rotamer outliers (%)	1	1	2	0
Clash score	12	20	22	17
Average B-factor (Å^2)	289.03	291.94	213.03	227.17

Supplementary Table 1. Cryo-EM data collection, data processing and model building statistics.

Supplementary table 2

**SERCA state**	PDB ID	$HMA4^{apo}$	$HMA4^{Cu}$	$HMA4^{AlF}$	$HMA4^{BeF}$
		**E1-apo**	**E1-Cu**	**E2P-AlF**	**E2P-BeF**
E1	4H1W	9.5	8.0	12.1	12.2
[Ca]2 E1	2C9M	10.9	11.8	14.5	14.5
[Ca]2 E1	1SU4	11.2	11.5	13.2	13.0
[Ca]2 E1×ATP	3N8G	10.9	8.2	12.1	12.6
[Ca]2 E1P-ADP	1T5S	11.0	8.3	12.1	12.6
[Ca]2 E1-ADP:AlF4	1T5T	11.0	8.2	12.1	12.6
[Ca]2 E1P:ADP	3BA6	10.9	8.2	11.8	12.3
E2P	3B9B	10.9	13.8	4.8	4.6
E2-P	3N5K	11.2	13.6	3.9	4.0
E2:Pi	3FGO	11.3	13.5	4.1	4.1
E2	3NAL	9.8	10.6	5.5	5.7
**P_{1B-1}-ATPases**					
$LpCopA^{AlF}$	4BYG	8.3	10.1	1.8	1.6
$LpCopA^{BeF}$	4BBJ	7.3	8.8	2.9	2.8
AfCopA	7R0I	4.1	3.8	10.8	11.4
XtATP7B	7SI3	7.7	9.7	1.7	1.8
hATP7B	7XUM	4.6	3.8	11.3	11.6
**AlphaFold models**					
HMA4		3.3	3.4	7.3	7.1
hATP7B		3.5	3.4	7.5	7.5

**M-domain**	$HMA4^{apo}$	$HMA4^{Cu}$	$HMA4^{AlF}$	$HMA4^{BeF}$
$HMA4^{apo}$		0.9	3.0	3.3
$HMA4^{Cu}$	0.9		3.0	3.1
$HMA4^{AlF}$	3.0	3.0		0.6
$HMA4^{BeF}$	3.3	3.1	0.6	

Supplementary table 2: RMSDs of structural alignments of the determined HMA4 structures to the available structures of relevant P-type ATPases. Alignments were computed using super in Pymol and using the complete structures, except where indicated.

Supplementary table 3

System	Box dimensions	Nr atoms	Nr waters	Salt, concentration	Lipid composition
E1^{apo}	x: 120.2 Å<br>y: 120.2 Å<br>z: 156.5 Å	211,256	48,416	KCl: 0.15 M	POPC: 383
E2P^{AlF}	x: 120.2 Å<br>y: 120.2 Å<br>z: 160.3 Å	218,306	50,648	KCl: 0.15 M	POPC: 388
E2P^{Xt}	x: 120.1 Å<br>y: 120.1 Å<br>z: 171.9 Å	343,561	92,435	KCl: 0.15 M	POPC: 387

Supplementary table 3: Simulation system overview. Specifications of simulation box dimensions, numbers of atoms, water molecules, salt concentration, and lipid composition of the membrane.

Supplementary table 4

System	Trajectories (samples)	Mean CA RMSD (Å)	Stdev (Å)
E1^{apo} MBD^{-2}	10	3.0	0.82
E2P^{AlF} MBD^{-2}	5	5.9	1.0
E2P^{Xt} MBD^{-2} all	5	9.8	8.1
E2P^{Xt} MBD^{-2} stable	3	3.5	0.87

Supplementary table 4: Calculations of MBD^{-2} root mean square deviations (RMSD). RMSD for MBD^{-2} was calculated compared to the initial position. The mean and standard deviation values presented were calculated from the mean RMSD of each replica. "E2P^{Xt} MBD^{-2} stable" refers to the three replicas where MBD^{-2} did not dissociate within the simulation time.

Uncropped SDS-PAGE gels - Supplementary figure 2

HMA4:

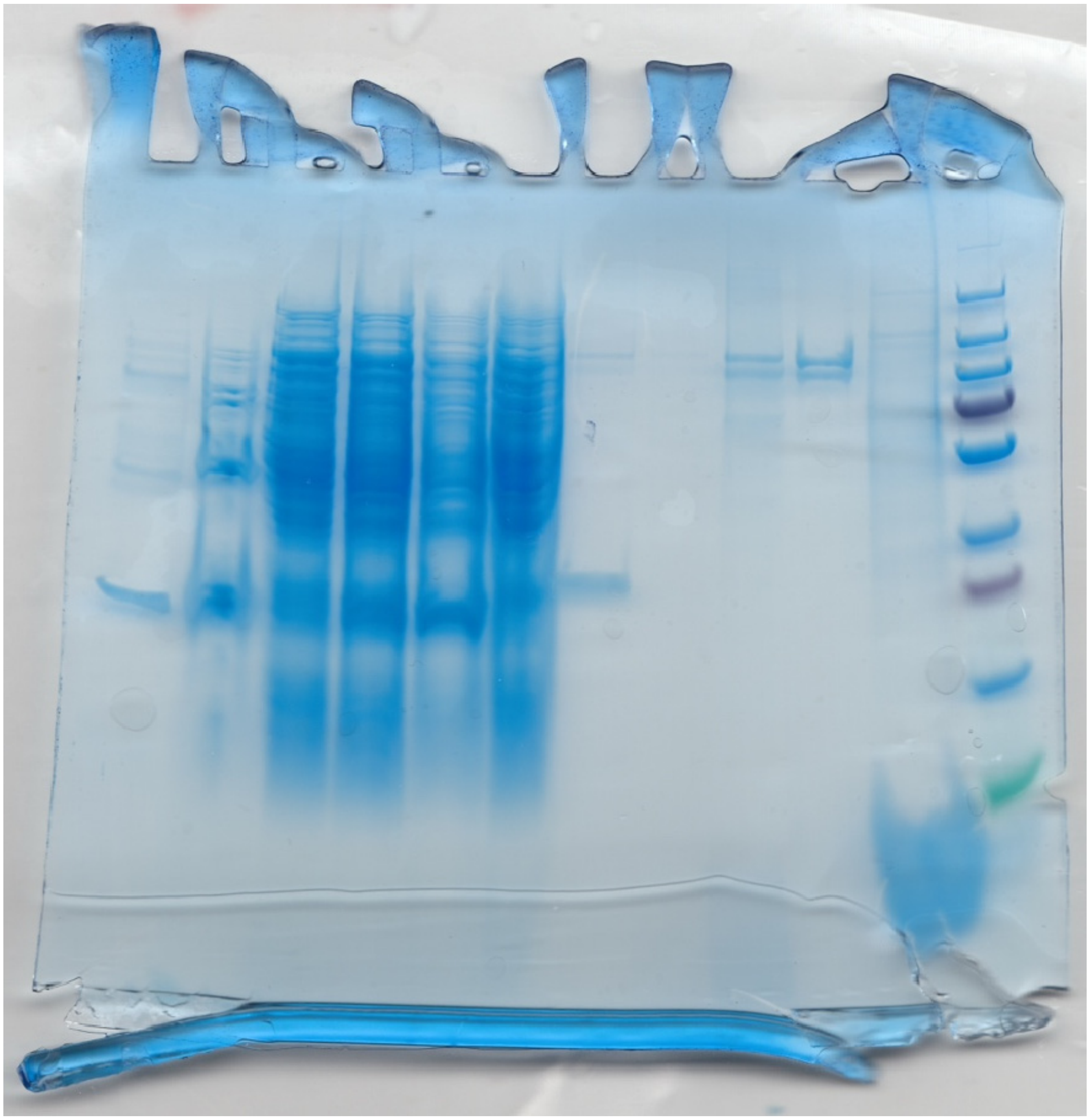

ATP7B:

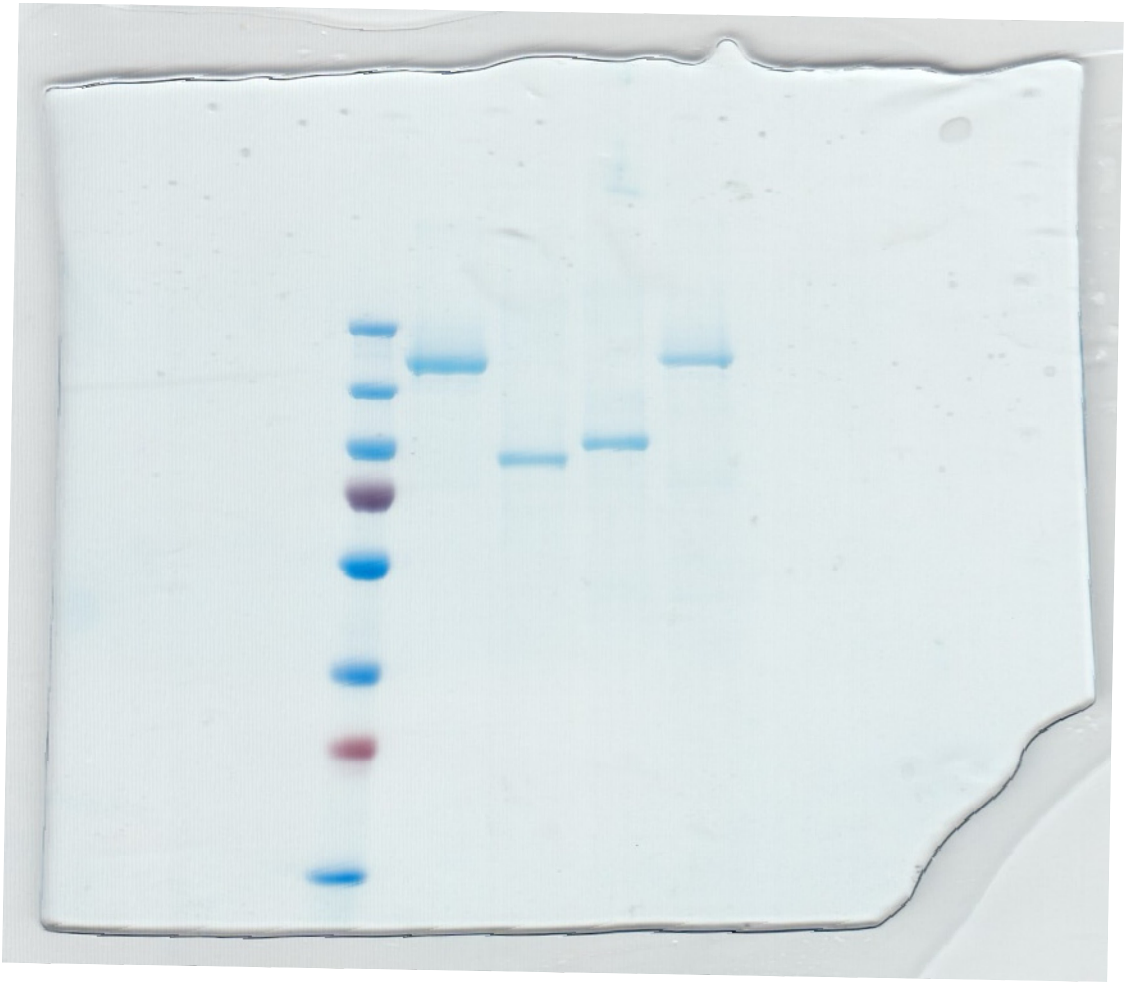

Supplement: Supplementary file 1 — Supplementary Info [file 41467_2024_47001_MOESM1_ESM.pdf]
