# Supplementary material for: Diverse roles of the metal binding domains and transport mechanism of copper transporting P-type ATPases
Source: Nat Commun. 2024 Mar 27;15:2690. doi: 10.1038/s41467-024-47001-4 (PMC10973460; doi:10.1038/s41467-024-47001-4)
Supplement: Supplementary file 3 — Source Data [file 41467_2024_47001_MOESM3_ESM.zip › 453495_2_related_ms_8739761_s9m909.docx]

**Source data file for:** **Diverse roles of the metal binding domains and transport mechanism of copper transporting P-type ATPases**

**Source data for Figure 3A, percent activity of WT for ATP7B**

|  | WT | WT AlF^4-^ | Δ1-6 | Δ1-6 AlF^4-^ | Δ2-6 | Δ2-6 AlF^-4^ |
| --- | --- | --- | --- | --- | --- | --- |
|  | 1 | 2 | 3 | 4 | 5 | 6 |
|  | 109.839927 | 4.05066055 | 11.6454523 | 10.3643024 | 50.2574447 | 10.070153 |
|  | 95.7595465 | 6.21246861 | 13.6090763 | 9.51294628 | 62.1848293 | 13.7555923 |
|  | 97.6051325 | 10.1430287 | 19.7458399 | 12.7466636 | 49.8752588 | 22.4113029 |
| Averages | 101.068202 | 6.80205263 | 15.0001228 | 10.8746374 | 54.1058443 | 15.4123494 |
| Std-dev | 7.65237954 | 3.08868005 | 4.22555651 | 1.67617521 | 6.99921542 | 6.33518971 |
